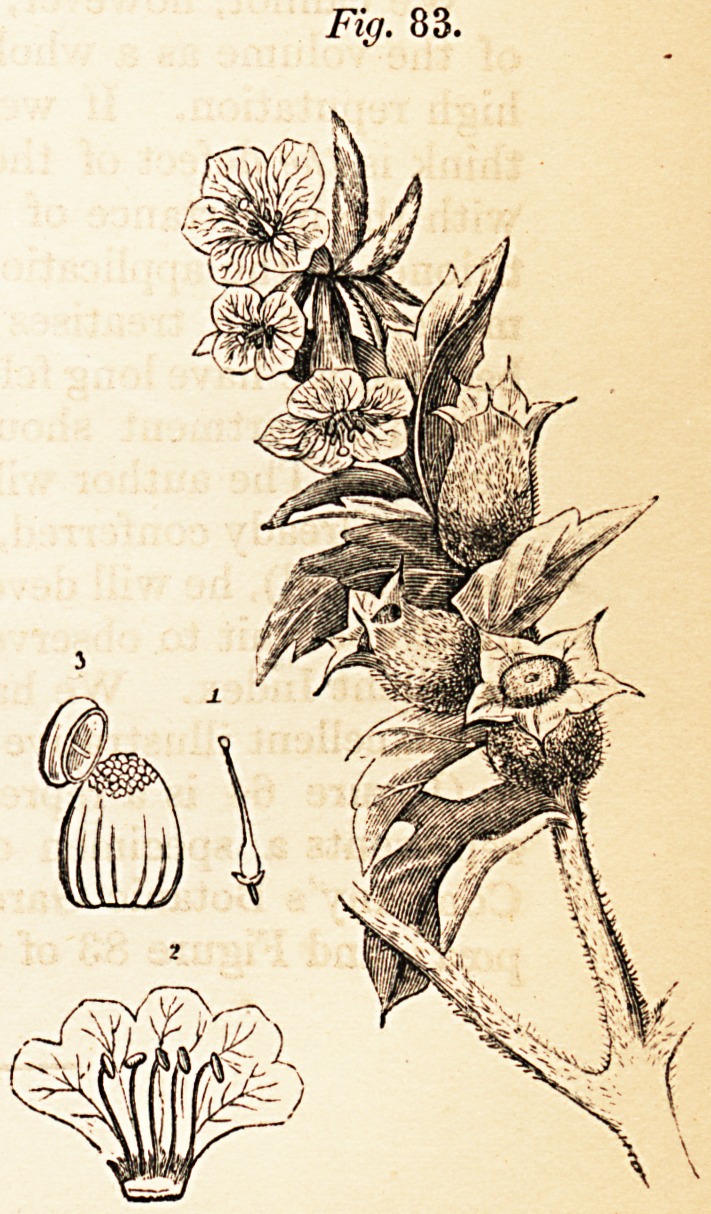# A Manual of Materia Medica and Therapeutics, Including the Preparations of the Pharmacopœias of London, Edinburgh and Dublin, with Many New Medicines

**Published:** 1847-01-01

**Authors:** 


A Manual of Materia Medica and Therapeutics, including the Preparations of the Pharmacopœias of London, Edinburgh, and Dublin, with many New Medicines. By *J. Forbes Royle*, M.D., F.R.S., &c. &c., Professor of Materia Medica and Therapeutics, King's College, London. Small 8vo, pp. 716. London: Churchill, 1846.

This volume is one of a very useful series of Manuals, beautifully got-up and published by Mr. Churchill. Although the form of the volume and the name "Manual" might induce some persons to anticipate any thing not like a complete and elaborate treatise on the subject, we can assure our readers that they have here as much matter as would fill two ordinary-sized octavo volumes, and a very full and complete treatise on Materia Medica; adapted not merely to the wants of students, for whom it is principally intended, but also to those of the practitioner. The beautiful paper and typography, and the excellent wood-cuts, are beyond all praise, albeit the smallness of the type, remarkably clear though it be, will not, we fear, find favour in the eyes of many, even of those who, like ourselves, have not yet seen three-score years.

In issuing so expensive a volume at so low a price, the publisher has, we doubt not, rightly estimated the wants of the profession, and the probable sale of such a work. "If it should be asked," says the author, "whether another work on materia medica was required in addition to the numbers which already exist, it must be replied, that this was undertaken at the repeated request of its publisher, who may be supposed to be well acquainted with the wants of the profession. This one, however, would not have been sufficient to induce the author to undertake the work, had he not also been aware from the complaints of pupils, and convinced from his experience as a teacher, that the student of materia medica required something systematic to study, which, brought up to the present time, should be sufficiently full for information, and yet as short and condensed as was compatible with the avoidance of being superficial." In both respects, although unable to satisfy himself, we think that, with one exception, to which allusion will subsequently be made, the author has been abundantly successful.

The difficulty experienced, both in the lecture-room and in preparing a work like the present, of treating many parts of the subject briefly, is, as the author states, increased, in consequence of the authoritative regulations by which students are compelled to attend the course of Materia Medica, during the first year of their attendance on lectures, ignorant of all the sciences, an acquaintance with which is absolutely necessary to the study of materia medica. It would doubtless be a great improvement in the existing regulations, if the student were compelled to commence his medical studies by Summer courses of Botany and Zoology, or Comparative Anatomy; Chemistry and Natural Philosophy having constituted a part of his preliminary education. As it is, there is a great deal of needless repetition in the several departments of our Colleges, and the subject of materia medica is rendered unnecessarily complex, and too often wearisome to the student. But there is also, in our estimation, a greater evil still. For, owing to the multiplicity of subjects of which the professor of materia medica and therapeutics has to treat, the most important department, the actions of medicines and their application to practical purposes, therapeutics, is treated as though it were of minor and subordinate importance. Yet this is the information which the student does not get elsewhere, and for which he naturally looks to the teacher of therapeutics.

Nor is this very serious evil confined to the lecture-room ; it obtains more or less in almost all our modern works on materia medica, and the present one, we are sorry to find, does not form an exception. Let it not be supposed that we are advocating mere theoretical disquisitions on the physiological or essential actions of medicines, of which, in nine cases out of ten, we may be said to know little or nothing. To students, at least, such speculations would be of little or no value, and often indeed worse than useless. But, to take as an illustration of our meaning, the first article on which we happen to open in the volume before us, Sulphate of Magnesia; surely, after reading through two pages of this very close print devoted to so common and useful a medicine, the student may fairly expect that its action and uses should not be dispatched in a single line, informing him that it is "cathartic, diuretic, a common constituent of a black dose!" And yet this is all that our author says to guide the student in the employment of one of our most common purgatives ;—he gives him not a word as to its character as a purgative, its advantages, or disadvantages, nor how it may be best prescribed. Or, we may take as another example, Ipecacuanha. The following is all that is said under the head of "actions and uses." "Irritant, Nauseant Emetic, Expectorant, Diaphoretic, Sedative. Useful as an Expectorant and Diaphoretic in Catarrh, or as a Diaphoretic in febrile affections of various kinds, or to cause a determination to the skin in Diarrhœa and Dysentery. Emetic to cut short the accession of an ague, &c., evacuate the stomach, or give a shock to the system. Nauseant Sedative in Hæmorrhage, &c." Now every practitioner knows that the action of Ipecacuanha as an Emetic is very different from that of Tartar Emetic or Sulphate of Zinc, and that in a given case it is by no means immaterial which is selected. The student, however, would not learn from his Manual that there was anything distinctive in the operation of these several emetics. For, under the head of Sulphate of Zinc, he is merely told that it is "in large doses Emetic;" and, on referring to the article Emetic Tartar, he simply learns that, in doses of "gr. j—ij. diluted," it is "emetic." No reference is made to the remarkable effects of Ipecacuanha on some constitutions in exciting paroxysms resembling asthma; nor to its very important therapeutic influence on the gastro-intestinal mucous membrane. From an Indian practitioner we might also have expected something more specific in reference to its sedative action and mode of exhibition in Dysentery. But the special therapeutic applications of the drugs are scarcely ever mentioned, or, at all events, are but alluded to in the most cursory manner. A sense of duty compels us to give prominence to this very serious defect, in the practical or therapeutical part of the present volume, by which its value to both student and practitioner is most materially diminished. As, however, it is almost the only fault we have to find with the work, we have had the less hesitation in expressing our disapprobation. For the rest, it has our hearty approbation. We proceed, therefore, to give our readers an account of Dr. Royle's arrangement and mode of treating his subject.

The first part of the volume is occupied by a brief Introduction, containing a sketch of the operations of Pharmacy, and of the principles of pharmaceutical chemistry. The Materia Medica is then arranged under three sections—mineral, vegetable, and animal. A natural-history arrangement is adopted throughout, and for the mineral department that which is employed in chemical works—proceeding from the non-metallic elements to the metals. The author does not confine himself to the articles which have a place in the English, Scotch, or Irish Pharmacopœias, but commences with Atmospheric Air, Oxygen, Hydrogen, and Nitrogen, and has introduced most of the new medicines. At the head of each article the synonyms are given, followed by the chemical symbols, and equivalents, the etymology, and a short account of the natural history. The physical and chemical properties are then described, the pharmaceutical preparations detailed, then follows the tests for their purity, their actions and uses, and, when necessary, their antidotes. The various formulæ of the several British Pharmacopœias, into the composition of which the different articles and their compounds enter, are appended, with brief explanatory remarks.

In the arrangement of the Vegetable Materia Medica the system of Decandolle is followed, and that of Professor Grant for the articles derived from the Animal Kingdom.

There is, as the author fears, some degree of obscurity in the mode in which the directions for making the preparations of the three Colleges are given, owing to the necessity which he felt himself under to condense these directions as much as possible. We subjoin the following as a specimen of the author's mode of treating the mineral department of his subject.

" FERRI SULPHAS, L. E. D.

" Sulphate of Iron. Sulphate of the Protoxide of Iron. *Ferrum Vitriolatum. Sal Martis. F.* Sulfate de Fer. *G.* Schwefelsaures Eisenoxydul. Eisenvitriol.

" Vitriolated Iron, or Green Vitriol, was known to the ancients. It is mentioned in the *Amera Cosha* of the Hindoos (*Hind. Med.* p. 44), and it is used by them, as by the Romans in the time of Pliny, in making Ink. It is found in nature: the Sulphuret, absorbing Oxygen from the atmosphere, is converted into the Sulphate of the Protoxide of Iron: this is apt to be changed into the red-coloured Sulphate of the Sesquioxide. The Sulphate, being soluble, is found in some mineral waters. It is also made artificially on a large scale for use in the arts, by exposing moistened Pyrites to the air, and is called Copperas or Green Vitriol.

" Sulphate of Iron ($FeO\ SO_3 + 7\ Aq. = 139$) is a transparent crystallized substance of a bluish-green colour, and a styptic (which is also called an inky) taste. The crystals are modifications of the oblique rhombic prism. Sp. Gr. 1·82. They are soluble in a little more than their own weight of cold and in ¾ of their weight of boiling water. In the air they effloresce, and the salt, absorbing Oxygen, is converted into the Sulphate of the reddish-coloured Sesquioxide of Iron. Heated, it is first melted in its water of crystallization; this is afterwards expelled, and the salt reduced to the state of a dry white powder. (*v.* Ferri Sulphas exsiccatus, E.) At a still greater heat, the acid is expelled, and may be obtained in the form of anhydrous or glacial Sulph′, the latter portion being decomposed. The Iron is left in the state of the reddish-coloured Sesquioxide, the *colcothar* of old authors and of the D. P. Sulph. Iron is insoluble in Alcohol; its solution in water reddens Litmus; its Iron is precipitated on the addition of alkalis, alkaline earths, and their Carbonates, by the former as a Hydrated Protoxide, and by the latter as a Carbonate, which is soon changed into the red Sesquioxide. q. v. With Ferrocyanide of Potassium, a white precipitate is formed with the pure Sulphate of the Protoxide, but a blue one if the Sesquioxide be present: the same change of colour ensues when the former precipitate is exposed to the air. A black precipitate (Gallate of Iron) is formed when the Sulphate containing any of the Sesquioxide is added to an infusion or tincture of Galls, or of any other astringent vegetable. Comp. Fe O 25·9+S′ 28·8+Aq. 45·3=100·

" *Prep.* Mix *Sulphuric*′ ℥xiv. (7 parts, D.) with *Aq.* Oiv. (60 parts, D.) add *Iron-filings* ℥viij. (*Wire* 4 parts, D.) apply heat, (and when the effervescence is over, L.) filter (through paper, D.) Set the liquor aside to crystallize, (after due concentration, D.) and then concentrate the supernatant liquor to obtain more crystals. Dry them all. (If the Sulphate of Iron of commerce be not in transparent green crystals, without efflorescence, dissolve it in its own weight of boiling water acidulated with a little Sul′; filter, and set the solution aside to crystallize. Preserve the crystals in well-closed bottles, E.)

" This process is introduced, as the Green Vitriol of commerce is usually impure. Concentrated Sul′ does not act on pure Iron, but the water of the dil. acid becoming decomposed, yields its O. to the Iron, while H. escapes in the form of gas. The Oxide of Iron formed unites with the Sul′, and the Sulphate of Iron is obtained.

" *Tests.* Pale bluish-green crystals, with little or no efflorescence; entirely soluble in water; this solution does not deposit Copper upon Iron being immersed in it; its solution, first boiled with Nit′ and then precipitated by excess of Ammonia, yields on filtration a fluid which is colourless or very pale blue. L. and E. If it be of a deep blue, then Copper is present. The boiling in Nit′ is not always necessary, for Green Vitriol is usually a mixture of Sulphate of Protoxide and of Sesquioxide of Iron. Zinc may be similarly detected by adding Ammonia in excess to the Sesquioxidated solution; after filtering, expel the excess of Ammonia by heat, and any Zinc which is present will be deposited in flakes of the white Oxide.

" *Inc.* Alkalis and their Carbonates, salts of Calcium and of Barium, Acetate and Diacetate of Lead, Nitrate of Silver, Vegetable Astringents.

" *Action. Uses.* Astringent, Tonic, Emmenagogue.

" *D.* gr. j.—gr. v. in pills with Bitter Extracts or Aromatic Confection.

" Mr. Phillips warns from giving it in solution without first boiling the water, and expelling its atmospheric air, of which the Oxygen would peroxidize the Oxide." P. 138.

In addition to the officinal preparations, we have a good account of most of the new preparations of this invaluable remedy.

The Vegetable Materia Medica is prefaced by a Botanical Introduction comprising a description of the parts of plants used officinally, a sketch of their "Classification" of "Vegetable Physiology," the "Geography of Plants," the connexion between the medical properties of plants and their structure or natural arrangement, and the collecting and drying of vegetables. To this introduction, as it is brief and will doubtless be useful to the student, no objection ought perhaps to be taken, on the score that some parts of it are out of place in a work on Materia Medica.

As might be expected, Dr. Royle's philological acquirements, and his acquaintance with the natural history of the East, have enabled him to throw much light on some obscure points connected with the etymology of many of the names of articles of eastern origin, as well as on their natural and commercial history. He has given, in his Preface, a list of the chief original authorities of which he has made use, both in the Botanical and Pharmaceutical department; but the attention which he has himself paid to the identification of officinal plants and the peculiar facilities which he has possessed for obtaining information from his contemporaries who have devoted themselves to similar investigations in the East, have enabled him to enrich this portion of the volume with much new and valuable matter. We may refer, in corroboration of this remark, to the very interesting and valuable article on Assafœtida, which may also be taken as an example of Dr. Royle's method of treating the Vegetable Department of the Materia Medica.

"ASSAFŒTIDA, L. E. D. Gummi Resina, L. D. Gummy-resinous Exudation (E.) of NARTHEX (*Ferula*, Linn.) ASSAFŒTIDA, *Falconer*. Assafœtida.

Assafœtida, a product of Persia and Affghanistan, is mentioned in the ancient Sanscrit Amera Cosha. The ancients highly esteemed a gum-resin which the Romans called *Laser*, and the Greeks ὀπος κυρηνακιὸς, or the Cyrenaic Juice, from being produced in that region. The plant σιλφιον yielding it was an Umbellifer, and is represented on the coins of Cyrene. It has been discovered of late years, and named *Thapsia Silphium*. This Laser had become scarce even in the time of Pliny, who as well as Dioscorides describes another kind as obtained from Persia, India, and Armenia, which was probably the same that was known to the Hindoos. Avicenna describes *hulteet* as of two kinds: one, of good odour, from Chiruana (Cyrene?), and the other fætid, the present *Assa-fœtida.* The term *assa* is no doubt of oriental origin, since it is applied to other gum-resins. Thus Benzoin is called *hussee-looban;* it used to be called *Assa dulcis* in old works. Dr. Lindley has received the seeds of a Ferula called *hooshee.* Anjedan, the fruits or seeds (φυλλον of the Greeks), is usually translated *Laserpitium.* The plant is called *Angoozeh* by the Arabs. The root of *Silphion* is described by Arrian as affording food to herds of cattle on Paropamisus.

Assafœtida is produced in the dry southern provinces of Persia, as in the mountains of Fars and of Beloochistan, but chiefly in Khorassan and Affghanistan; likewise to the north of the Hindoo Khoosh range of mountains, where it was found by Burnes and also by Wood's expedition to the Oxus (*c.*) Dr. Falconer found it in Astore, introduced the plant into the Saharunpore Botanic Garden, as mentioned in the author's 'Product. Resources of India,' p. 223, and has obtained from it a small quantity of Assafœtida. He also sent home numerous seeds, which were distributed from the India House to several gardens; but the author has not heard whether any plants have been produced from them. But he has no doubt that some of those which the author is informed by his friend Dr. Christison are still in the Edinburgh Botanic Garden, were produced from these seeds, and not from those sent by Sir John M'Neill. The Assafœtida is conveyed on camels into India across both the Punjab and Bhawulpore, and is sold in large quantities at the Hurdwar Fair. It is also conveyed down the Indus and by the Persian Gulf to Bombay.

"Two or three kinds of Fruit called Seeds are met with, which are said to be those of the Assafœtida plant, but there is no proof that more than one plant yields Assafœtida. Dr. Falconer, an excellent Botanist, after examining the original specimens, considers the plant he saw in Astore to be the same as that figured by Kæmpfer; and Dr. G. Grant, who saw the plant at Syghan, says, as stated by Dr. Christison, that its roots, leaves, and flowering stem correspond on the whole with Kæmpfer's description, except that the root is deeply divided, like the outspread hand. The E. P. assign *Ferula persica* as probably yielding some Assafœtida. There is no doubt that its seed has been sent from the north-west of Persia as those of the Assafœtida plant; but there is no proof, nor indeed is it probable, that it yields any of the Assafœtida of commerce. The gum-resins of these Umbelliferæ are too similar to each other, for any but experienced pharmacologists to determine between *inferior* Assafœtida and varieties of Sagapenum or other Gum-resins.

" As Dr. Falconer, the author's friend and successor as Superintendent of the East India Company's Botanic Garden at Saharunpore, has had excellent opportunities for examining the Assafœtida plant, both in its native sites and as cultivated by himself, he has favoured the author with the following full account of this important plant, which he conceives belongs to a genus allied to but distinct from Ferula." P. 414—15.

Dr. Falconer's elaborate botanical description of the plant we are compelled to omit; he, however, is satisfied that what he describes is the true " Assafœtida disgunensis" of Kæmpfer, which does not appear to have been met with by any other botanist since it was examined *in situ* by that excellent observer, a century and a half ago. Both in the characters of the flowers, and fruit, and in its " Pæony-leaved" habits (the true Assafœtida plant) differs widely from any known species of Ferula and appears to constitute a distinct and well-marked genus, to which Dr. Falconer has given the name of *Narthex*, from the Greek νάρθηξ. This is the word employed by Dioscorides to designate an umbelliferous plant which in Latin is called Ferula, the name which Linnæus adopted for the genus to which the Assafœtida plant was formerly supposed to belong. Our classical readers will recognize νάρθηξ as the plant in whose pithy stalk Promœtheus is said to have conveyed to earth the fire of heaven. The Greeks, in the present day, employ the pith for the purposes of tinder and as a means of carrying about fire. The stalks likewise furnished the Bacchanalian wands and splints for bandaging broken limbs.

" In the Dardoh or Dangree language (the Dardohs being the Daradi of Arrian) the plant is called ' Sip' or ' Sup.' The young shoots of the stem in spring are prized as an excellent and delicate vegetable.

" The species would appear to occur in the greatest abundance in the provinces of Khorassan and Laar in Persia, and thence to extend on the one hand into the plains of Toorkestan on the Oxus north of the Hindoo Khoosh mountains, where it seems to have been met with by Sir Alex. Burnes, and on the other to stretch across from Beloochistan, through Candahar and other provinces of Affghanistan to the eastern side of the valley of the Indus, where it stops in Astore, and does not occur in great abundance. The whole of this region, which constitutes the head-quarters of the gum-bearing Umbelliferæ, possesses the common character of an excessively dry climate, indicated in Berghaus's hygrometric map in Johnson's Physical Atlas by a belt of white.

" Besides the gum-resin, the fruit of *Narthex Assafœtida* is imported into India from Persia and Affghanistan, under the name of ' Anjoodan,' being extensively employed by the native physicians in India: ' Anjoodan' being the epithet applied to the seed of the ' Heengseh,' or ' Hulteet,' by Avicenna, also quoted by Kæmpfer, and used by the Indo-Persian and Arabic writers generally in describing the Assafœtida plant." P. 419.

The following account of a new remedy which is likely to come into more extensive use will be interesting to our readers.

"Bebeerine. Alkali of Nectandra Rodiei, *Schomburgk.* Greenheart Tree.

"A considerable quantity of a wood called Greenheart is imported into this country for ship-building. It is large in size, heavy, hard, durable, takes a polish, but is apt to split, and is of different tints of olive-green, varying from pale to dark.

"Sir R. Schomburgk, Hooker's Journ. of Bot. Dec. 1844 (British Assoc. 1845), has described the tree which yields the *Greenheart* timber of Guiana (called *Bebeera* by the Indians of Demerara, and *Sipeeri* by the Dutch colonists). It is a new species of the Laurels, belonging to the genus *Nectandra,* and which has been named *N. Rodiei,* in compliment to Mr. Rodie, late a surgeon in the R. N., who first, in 1834, directed attention to its valuable febrifuge properties and indicated the presence of an alkali in the bark of this tree. Dr. Warburg also prepared what he called 'Vegetable Fever Drops' from some part of this tree, which he distributed extensively, and which were favourably reported on by various medical officers. Dr. Maclagan in April 1843 read before the Royal Society of Edinburgh an able paper on the Bebeera Tree, its chemical composition, and its medical uses; and the nature of the alkali Bebeerine has been further elucidated by himself and T. Tilley, Esq. Professor of Chemistry in Birmingham, in a paper read before the Chemical Society. The medical virtues of this alkali, or rather, of its Sulphate, have been detailed by Dr. Maclagan, &c. in the Lond. and Ed. J. of Med. Science, July 1843 and April 1845.

"The bark of the Bebeera tree occurs in large flat pieces, is about four lines in thickness, heavy, and with a rough fibrous fracture, of a dark cinnamon-brown colour, rather smooth within, but covered externally by a splintering greyish-brown epidermis. It has little or no aroma, but a strong, persistent, bitter taste, with considerable astringency. These properties depend on the presence of an alkali, which has been called Bebeerine. Dr. M. at first thought that there were two alkalis; but this, from his second paper, does not appear to be the case. It is contained also in the seeds, as is evident from Dr. M.'s analysis of both the bark and seed.

| | *Bark.* | *Seeds.* |
|---|---|---|
| Alkalis (not quite pure) | 2·56 | 2·20 |
| Tannin and Resinous matter | 2·53 | 4·04 |
| Soluble matter (Gum, Lignin, Salts) | 4·34 | 9·40 |
| Starch | 0· | 53·51 |
| Fibre and Albumen | 62·92 | 11·24 |
| Ashes (chiefly Calcareous) | 7·13 | 0·31 |
| Moisture | 14·07 | 18·13 |
| Loss | 6·45 | 1·17 |
| | 100·00 | 100·00" P. 546. |

Messrs. Maclagan and Tilley, to whom we are indebted for the investigation of this subject, observe that it is remarkable that Bebeerine should be isomeric with Morphia, which acts as a pure Narcotic. The constitution of the two bodies is identical, and these gentlemen hence conclude "that similarity of physiological properties does not depend upon similarity in the properties of their constituents. It seems probable that the mode in which their atoms are grouped has an important share in modifying their physiological actions." The actions and uses of this new alkaloid are (at a most unusual length) described as;—

"Tonic, Antiperiodic, Febrifuge. From the original experiments of Mr. Rodie, and those made with Warburg's Fever Drops, there was little doubt of the Bebeera bark being a powerful Antiperiodic. These have been confirmed by the experiments of Dr. Maclagan, and of Dr. Watt of George Town, Demerara, with the Sulphate of Bebeerine, and of Dr. Anderson and others at Kamptee, &c., in the Ague and Remittent Fever of India, by Drs. Bennett and Simpson, in Periodic Neuralgia. Dr. Christison has stated to the author that the Sulphate of Bebeerine has come into general use in Edinburgh as a Tonic and Stomachic, and also as an Antiperiodic, in the very same diseases and for the very same purposes, as Sulphate of Quinine, and that it appears not so apt to occasion headache. He had employed it in a very severe case of periodic Tic douloureux, and with complete success, exactly as if Sulphate of Quinine had been used. It is given in 2 or 3 grain pills every hour, or three or four times a day, according to the case, so that ℈j. or so, may be given before the accession of a paroxysm, or it may be given in gr. x. doses, morning and evening. Considerable improvement in the manufacture has been made by Mr. M'Farlane of Edinburgh, who now prepares it in considerable quantities for medical use in the form of the Sulphate of Bebeerine." P. 547.

Although we own ourselves to be of the number of those who are slow to put faith in the alleged virtues of new remedies, we think there is already sufficient evidence in favour of the tonic and antiperiodic virtues of Bebeerine to justify more extended trials.

With respect to another new remedy, the Canabis Indica, we believe that the general opinion of those who have tried it, in this country, would be, that it has failed to justify the eulogiums with which it was introduced into British practice. As, however, its remedial virtues are still exciting considerable interest and attention, we extract the following account of the plant and the various preparations of it.

"CANNABIS SATIVA and its variety *C. indica.* The Leaves and Resin of Hemp.

"The Hemp appears to be a plant of the Persian region, where it is subjected to great cold in winter, and to considerable heat in summer. It has thus been able to travel on one hand into Europe, and on the other into India; so that the varieties produced by climate have by some been thought to be distinct species, the European being called *C. sativa,* and the Indian *C. indica.* The name *καvvαβιs*, by which it was known to the Greeks, seems to be derived from the Arabic *kinnub,* the *canape* of the middle ages, Dutch *kinnup* and *hinnup,* German *hanf,* whence the English *hemp.* Herodotus mentions it as Scythian. Biberstein met with it in Tauria and the Caucasian region. It is well known in Bokhara, Persia, and abundant in the Himalayas. It seems to have been employed as an intoxicating substance in Asia and Egypt from very early times, and even in medicine in Europe in former times, as we find it noticed in Dale (*Pharmacologia,* i. 133) and Murray (*Apparat. Medicaminum,* iv. p. 608—620), where it is arranged, as in this work, next to the Humulus. It has of late years been brought into European notice by Dr. O'Shaughnessy.

"The Indian plant has by some been thought to be a species distinct from the European one; but, like Dr. Roxburgh and others, the author was unable, when in India, to observe any difference between the plant of the plains and that of the hills of India, nor between these and the European plant. The Indian secretes a much larger proportion of resin than is observable in the European plant, but a difference is observed in this point in India between plants grown in the plains, and those of the mountains, and also when grown thickly together. The natives plant them wide apart, to enable them to secrete their full powers. In Europe, the thick sowing, and moister, often dull, climate will prevent the due secretion of the peculiar principles of a plant of the Persian region. But the plants grown in the past season, from the great heat and light, ought to be more resinous than usual. It is not without interest to observe that both the Hop and Hemp, belonging to the group *Cannabineæ*, owe their properties to the glandular resinous secretions. The author, in calling attention to the uses of this plant, in his *Illustr. of Himalayan Botany*, stated that 'the leaves are sometimes smoked in India, and occasionally added to Tobacco, but are chiefly employed for making *bhang* and *subzee*, of which the intoxicating powers are so well known. But a peculiar substance is yielded by the plants on the hills, in the form of a glandular secretion, which is collected by the natives pressing the upper part of the young plant between the palms of their hands, and then scraping off the secretion which adheres. This is well known in India by the name of *churrus*, and is considered more intoxicating than any other preparation of the plant; which is so highly esteemed by many Asiatics, and serves them both for wine and opium: it has in consequence a variety of names applied to it in Arabic, some of which were translated to me, as 'grass of faqueers,' 'leaf of delusion,' 'increaser of pleasure,' 'exciter of desire,' 'cementer of friendship,' &c. Linnæus was well acquainted with its 'vis narcotica, phantastica, dementens' (anodyna et repellens). It is as likely as any other to have been the *Nepenthes* of Homer."* P. 570.

Dr. O'Shaughnessy has described the different preparations as—1. *Churrus*, the concreted resinous exudation from the leaves, slender stems and flowers. 2. *Ganjah.* Bundles about 2 feet long, containing 24 dried plants, which have flowered, but from which the resin has not been removed. 3. *Bang*, *Subjee*, or *Sidhee*, formed of the larger leaves and capsules without the stalks.

"All these preparations are capable of producing intoxication, whether the *churrus* be taken in the form of a pill, or with conserve, or the dried leaf be rubbed up in milk and water with a little sugar and spice, or smoked. As a medicine, it was tried by Dr. O'S. in Rheumatism, Hydrophobia, Cholera, and Tetanus. In the last such marked benefit and cures were produced, that the Hemp was pronounced an Anticonvulsive remedy of the greatest value. Its general effects are, alleviation of pain (generally), remarkable increase of appetite, unequivocal Aphrodisia, and great mental cheerfulness. Its more violent effects were, delirium of a peculiar kind, and a cataleptic state. Dr. Pereira was among the first to submit it to experiment, but failed in obtaining any results, probably from changes having taken place in the drug. Dr. Laurie pronounced it uncertain, and not to be trusted to as a narcotic. Mr. Ley, however, found it useful in relaxing spasm, producing sleep, and during its action abatement of pain. Mr. Donovan found its power great in temporarily destroying sensation, and subduing the most intense neuralgic pain. Professor Miller of Edinburgh considers its virtue to consist in a power of controlling inordinate muscular spasm. Dr. Clendinning says that in his hands its exhibition has been followed by manifest effects as a soporific or hypnotic in conciliating sleep, as an anodyne in lulling irritation, as an antispasmodic in checking cough and cramp, and as a nervous stimulant in removing languor and anxiety. The Hemp may be used in the following preparations (Extract and Tincture) and doses: but Dr. O'S., when in England, found that he was obliged to give as much as 10 or 12 grs. or even more: though in India he considered gr. ½ a sufficient, and 1½ gr. of the Extract a large dose." P 571.

---

* "Dr. O'S. states that 'no information as to the medicinal effects of Hemp exists in the standard writers on Materia Medica to which we have access.' It is only in the later writers that it is omitted. Linnæus was acquainted with them, as the author quoted in the above briefly, as being a botanical work."

Whatever may be its virtues in other diseases of the nervous system, it has certainly failed most signally in all the cases of tetanus in which it has been tried in the London hospitals. And in several instances these trials were made with the extract prepared and brought to this country by Dr. O'Shaughnessy, and given, we believe, under his superintendance. The difference, therefore, in the effects of the remedy as exhibited here and in India, must, we think, be, in part, at least, attributed to difference of constitution, as the result of difference of climate. There are, it is well known, analogous differences in the effects of other narcotics.

We may refer to the article Cinchona for a specimen of Dr. Royle's ability and success in treating a difficult and complicated subject. The botanical account and commercial history of the various kinds of Bark, as well as their distinctive characters, though brief, are exceedingly luminous, and we believe as correct as the present state of our knowledge admits. But the brief paragraph treating of the action and uses of so important a remedy as Bark, is, we are compelled to say, in striking contrast with the other portions of this article. It could scarcely have been more meagre and unsatisfactory.

In the Animal Materia Medica there is nothing that appears to demand special notice. The volume, however, closes with a very useful Appendix, in which all remedies that may be used for the same therapeutical purposes are grouped together. To each group are prefixed some general observations on the nature and actions of the different classes of remedies. These the author states, in his Preface, should be read in connexion with the notices on the actions and uses of drugs at the end of each article. This division of his subject, the most important of the whole, it is very properly observed, would, if treated fully, require a volume to itself, and the apology for treating it so briefly, is the necessity for compressing the materials within the compass of a manual. But we cannot, after what we have already said, admit the apology. For valuable and useful as this Appendix is, it by no means atones for the seriously defective mode in which the therapeutic part is treated in the body of the work. Sixty, instead of thirty, pages might have been well devoted to this portion of the subject, and would have enabled the author, in some measure, to make amends for his one grand fault. But we should still have asked for more specific directions to guide the student in the use of particular remedies. To enable our readers to judge whether the strictures we have already made are rendered nugatory by the information afforded under the head of Emetics, we subjoin, as our last extract, the whole article.

"EMETICS.

"Medicines which evacuate the stomach by vomiting: an act produced partly by the influence produced on the stomach, and partly by that induced by the brain and nervous system. The latter we see in Sea-sickness, and the want of it in the difficulty with which Emetics act in narcotic poisoning, when the brain is in a comatose state. Emesis is also produced by tickling the fauces with a feather. Emetics differ much among themselves, some acting only when introduced into the stomach; others, as Tartar Emetic, if applied to any other part of the body, so as to be absorbed into the system. The effect is not altogether dependent upon the nature of the substance, for Ammonia and Mustard, which in small doses act as Stimulants, and Sulphates of Zinc and Copper as Tonics, will in large doses evert the action of the stomach, and produce an emetic effect, generally quickly, and without debilitating the system. Others act more slowly, and produce long-continued nausea, with the depressing symptoms which accompany such a state, and which are known to favour absorption. These are, therfore, as well as from their slow action, not suited to cases of poisoning. With both the act is accompanied by a series of concussions which favour the excretion and secretion of the biliary, pancreatic, and intestinal fluids, causing a determination to the skin. But this very concussion makes them dangerous when there is a determination to the head, or in advanced stages of pregnancy, in hernia, &c. But it makes them useful before the accession of an Intermittent, also in bilious Fever, likewise in Asthma, Hooping-cough; or they may be used for merely evacuating the stomach.

" *Direct Emetics, acting quickly.*

" Ammoniæ Liq. 58. Ammoniæ Sesquicarb. Liq. 64 (f℥ss.—f℥j. of either taken in a glass of cold, followed immediately by some warm water). Sodii Chloridum, 96, or common Salt is usually readily available.

" Zinci Sulphas, 151. Cupri Sulph. 154. Cupri Ammonio-Sulph. 155. Ærugo, 157.

" Sinapis nigra, 274. S. alba, 276.

" *Indirect Emetics.*

" Antimonii et Potassæ Tartr. (Tartarum Emeticum, D.), 177. Vinum, 180.

" Antimonii Oxidum, E. 172. Sesquisulphuret. et Oxysulphuretum, 175, 176.

" Ipecacuanha, 433. Pulv. Vin. et Syr. 436. Emetine, 435. Viola odorata, 278.

" Scilla. Pulv. Tinct. et Syr. 594, 595. Asarum, 548. Euphorbium, 558, but is too acrid.

" Anthemis, Inf. et Dec. comp. 458 : assists vomiting.

" Tabacum, 518. Lobelia inflata, 467 ; but both are unsafe as Emetics.

" Ipecacuanha and Tartar Emetic are often combined together, or the latter may be prescribed with a Cathartic, forming an Emeto-Cathartic." P. 675.

We cannot, however, conclude without expressing our warm approbation of the volume as a whole. It will certainly not detract from the author's high reputation. If we have expressed in very decided terms what we think is *the* defect of the volume, it is because we are deeply impressed with the importance of rendering every facility to both student and practitioner in the application to the purposes of daily practice of the information which treatises on Materia Medica are intended to convey, and because we have long felt it to be a crying evil that in such works, the therapeutic department should be neglected for mere chemical and botanical details. The author will, we are quite sure, add greatly to the obligations he has already conferred, if, in a future edition (which will undoubtedly soon be required), he will devote more space to the therapeutic department. We must not omit to observe that the utility of the volume is enhanced by an excellent Index. We have given a few of the drawings as a specimen of the excellent illustrative wood-engravings.

(Figure 64 is a representation of the Melaleuca Cajuputi; Figure 69 represents a specimen of Narthex Assafœtida, grown in the H. E. India Company's Botanic Garden at Saharunpore; Figure 78 of the Olea Europœa; and Figure 83 of the Hyoscyamus Niger.)

*Fig. 64.*

*Fig. 69.*

*Fig. 78.*

*Fig. 83.*

## Figures and Tables

**Figure f1:**
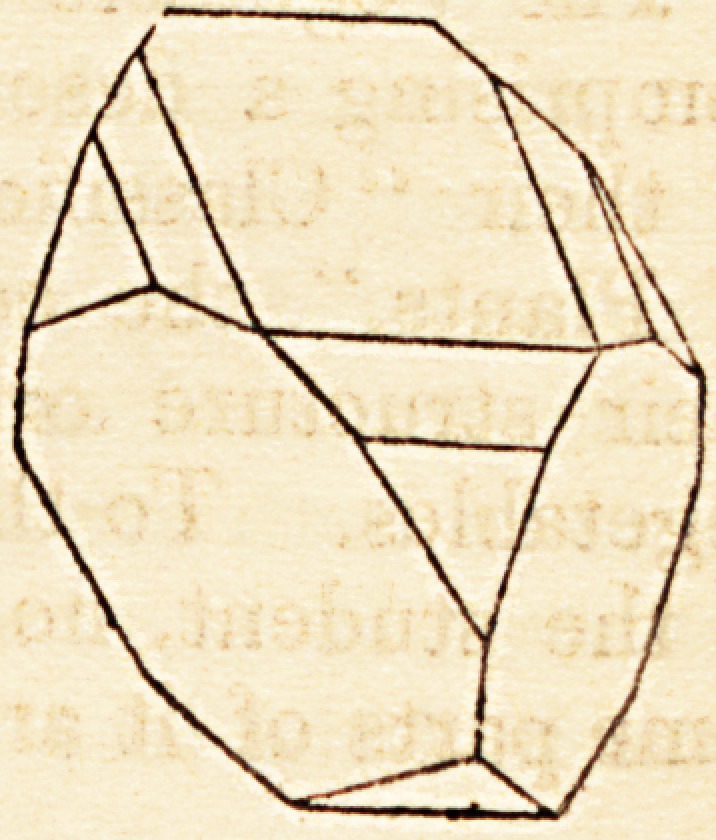


**Fig. 64. f2:**
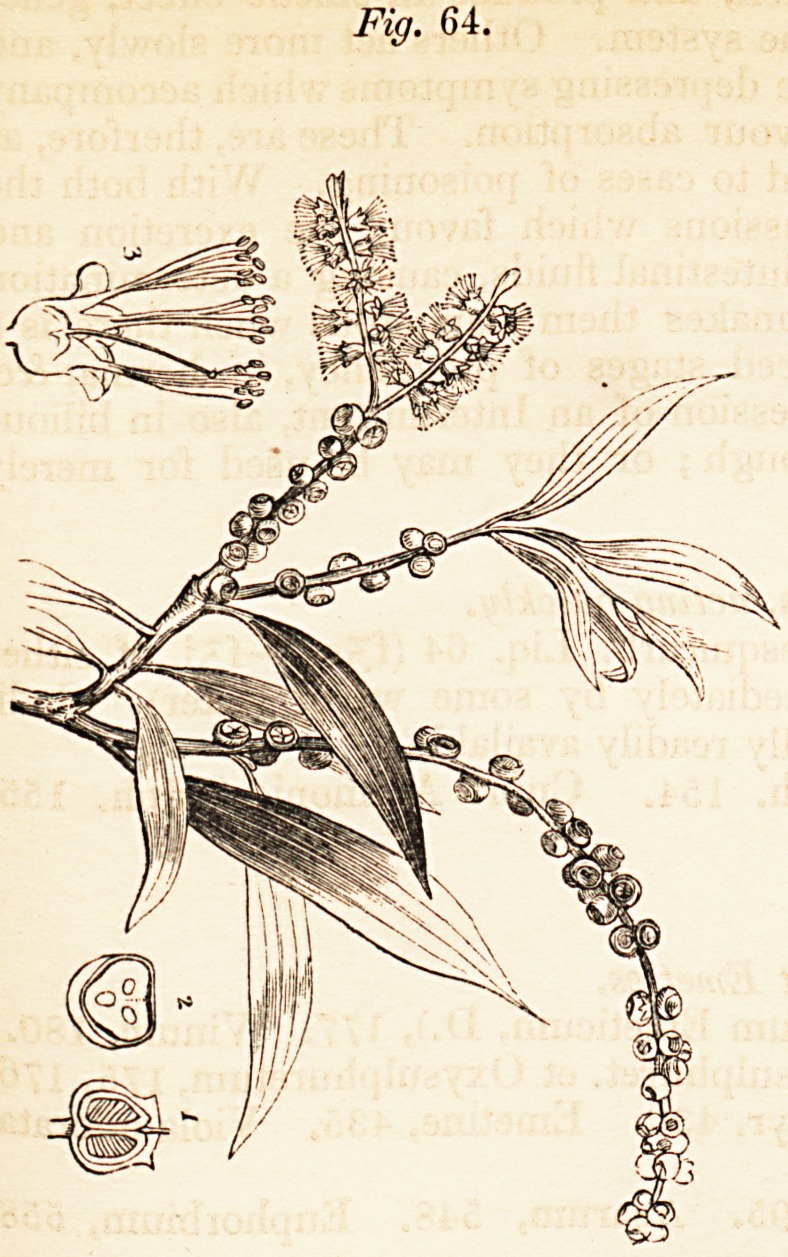


**Fig. 69. f3:**
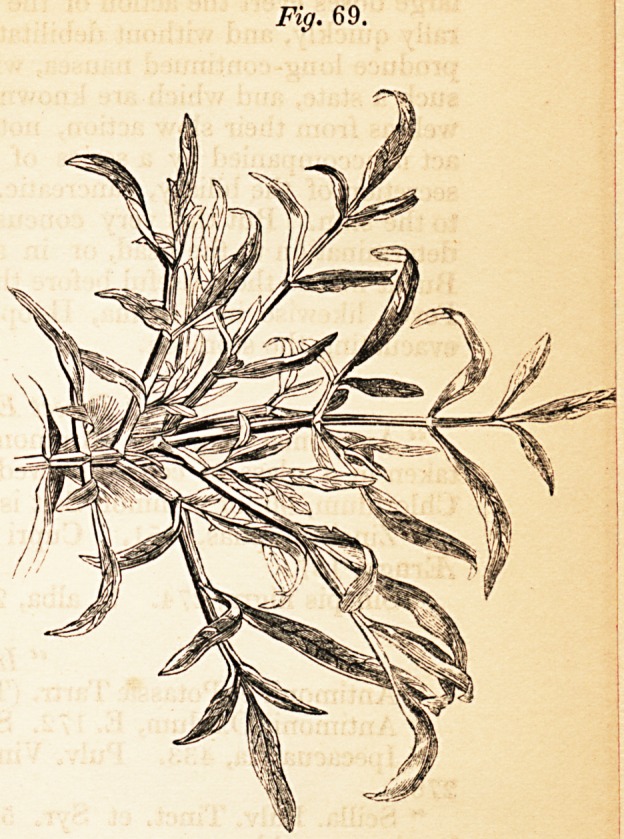


**Fig. 78. f4:**
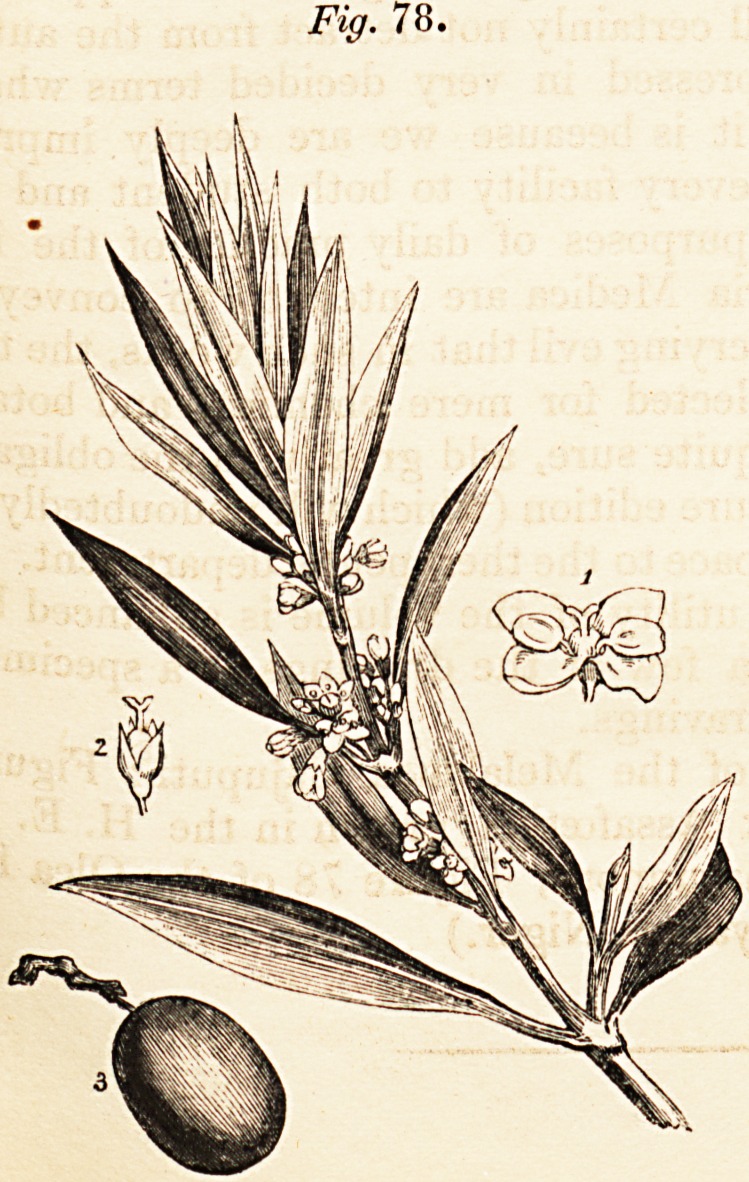


**Fig. 83. f5:**